# Non-Maleficence toward Young Kidney Donors: A Call for Stronger Ethical Standards and Associated Factors in Multidisciplinary Nephrology Teams

**DOI:** 10.3390/nursrep14030149

**Published:** 2024-08-19

**Authors:** Mahdi Tarabeih, Wasef Na’amnih

**Affiliations:** 1 School of Nursing Sciences, The Academic College of Tel-Aviv-Yaffa, Tel Aviv 64044, Israel; wasefna@mta.ac.il; 2 Department of Epidemiology and Preventive Medicine, School of Public Health, Sackler Faculty of Medicine, Tel Aviv University, Tel Aviv 69978, Israel

**Keywords:** living donation, autonomy, disclosure, ethical competence, non-maleficence

## Abstract

Background: The rising frequency of live kidney donations is accompanied by growing ethical concerns as to donor autonomy, the comprehensiveness of disclosure, and donors’ understanding of long-term consequences. Aim: To explore donors’ satisfaction with the ethical competence of multi-professional nephrology teams regarding disclosure of donation consequences to live kidney donors. Methods: A cross-sectional study was performed among Israeli live kidney donors who had donated a kidney in two hospitals that belonged to the Ministry of Health’s Transplantation Center one year after the donation, from December 2018 to December 2020. Data collection was conducted online and through face-to-face interviews with the donors in their native language (Hebrew or Arabic). Results: Overall, 91 live kidney donors aged 18–49 years were enrolled. Of those, 65.9% were males, and 54.9% were academic donors. Among the live kidney donors, 59.3% reported that the motivation behind the donation was a first-degree family member vs. 35.2% altruistic and 5.5% commercial. Only 13.2% reported that the provided disclosure adequately explained the possible consequences of living with a single kidney. Approximately 20% of the participants reported that the disclosure included information regarding their risk of developing ESRD, hypertension, and proteinuria. The donors reported a low mean of the index score that indicates a low follow-up by the physician after the donation (mean = 1.16, SD = 0.37). The mean GFR level was significantly lower in the post-donation period one year following a kidney donation (117.8 mL/min/1.73 m^2^) compared with the pre-donation period (84.0 mL/min/1.73 m^2^), *p* < 0.001. Conclusion: Our findings display that donors’ satisfaction with the ethical competence of multi-professional nephrology teams regarding the disclosure of donation consequences to live kidney donors is low. This study indicates that donors are at an increased risk of worsening kidney functions (creatinine and GFR), and BMI. Our findings underscore the imperative to advise donors that their condition may worsen over time and can result in complications; thus, they should be monitored during short and long-term follow-up periods. This study was not registered.

## 1. Introduction

Ethical competence (EC) is closely associated with an ethos of caring, as demonstrated in attitudes as well as actions, i.e., strength of character, ethical awareness, moral acumen, and a willingness to do good [1,2,3]. It is crucial to avoid ethically ambiguous actions. To date, most studies reporting on EC have focused on the many ethical decision-making models used when treating patients in various contexts (e.g., dementia, and hospitalized patients) [4,5,6,7]. The extant literature reporting on EC in multi-professional teams is scant [8,9]. Previous studies have acknowledged the need for more research on EC in multi-professional teams [1,3,10]. Moreover, the subject of EC as it relates to live kidney donors has been understudied, particularly regarding the responsibility of multi-professional teams to the donors whom they treat [11].

### 1.1. Live Kidney Donations

Ethical conflicts are often linked to issues in communication and teamwork, such as leaving employment which physicians see as opposed to their values, or even leaving the profession entirely. Studies have identified a positive correlation between ethical conflicts and occupational burnout and decreased job satisfaction [12,13,14]. Organ donation is an altruistic act that saves the recipient’s life, provides a sense of purpose and fulfillment to the donor, and helps the state reduce the transplant waiting list. It is important to distinguish between organ donation to a family member, which has a clear and known rationale, and altruistic donation to a non-family member, as opposed to donation within a donor exchange program [15]. There is an ethical issue in Israel that may jeopardize donors’ autonomy due to social pressure and coercion, with concerns about personal integrity being at risk because of the possibility of direct donation, which is allowed under Israeli law [16]. Thus, ethical issues regarding living kidney donors may be a greater concern in Israel, where long-term monitoring and ethical considerations for the donor’s health are under stricter scrutiny due to a religious and cultural environment that may discourage live donation and demand a more rigorous after-care for donors.

The practice of altruistic donation, despite the ethical debate, has achieved success and popularity. The ethical and legal foundation for living organ donation is based on the noble act of the donor, who is willing to compromise their bodily integrity and even risk themselves for the sake of another person in need of the organ, without any compensation. Nonetheless, the act raises an ethical dilemma (a clash of values) when the donor imposes conditions on their donation [17]. The process varies significantly between transplant centers, leading to confusion, misconceptions, and a lack of motivation among potential donors. Therefore, the donor evaluation, allocation, and post-donation follow-up processes must be standardized globally. A rigorous assessment protocol, including a mental health evaluation and informed consent, is essential for successful implementation. Promoting the practice of altruistic donation requires public awareness of its benefits, minimizing the risks to donors, education, and the involvement of all relevant stakeholders. This approach has the potential to increase transplantation rates and alleviate organ shortages, and thus it should be encouraged and managed carefully to shorten waiting lists, while ensuring no harm is caused to donors [17].

The legal and ethical dimensions of living kidney donations have been an issue of considerable debate throughout the extensive history of organ transplantation. Whereas there is broad agreement that transplantation represents the optimal treatment for end-stage renal disease (ESRD), concerns as to donor safety, motivations behind the donation, the imperative to do no harm, potential exploitation, and the specter of unbridled commercialism have led to variability in its acceptance and practice worldwide. Furthermore, some transplant teams have outright rejected this practice. Despite these challenges, there has been a remarkable surge in live kidney donation, which now comprises nearly half of all kidney transplants conducted globally. Live kidney donation represents a significant testament to the value placed on human life, although it introduces potential physical risks to individuals who are otherwise healthy, without providing any direct medical benefits to the donor. Ethically, the rationale for live kidney donation rests upon a decisive and substantial preponderance of benefits for both the donor and the recipient, relative to the risks entailed by the transplantation process from a living donor.

Live kidney donors attribute moral virtue, benevolence, and beneficence to clinicians [18]. There is an obligation-based accountability between the nephrology team and the living kidney donor who is awaiting a kidney transplant that draws on the focal virtue of discernment, entailing the ability to deliberate and reach decisions without being influenced by extraneous considerations [7,19].

Clinicians’ obligations of non-maleficence towards the live kidney donor are more stringent and override beneficence to the kidney recipient. Acts of non-maleficence respect autonomy and beneficence, and their implementation is critical in preserving public trust in clinicians, which has been shaken due to unethical practices [20]. When strangers meet in medical settings, medical codes of ethics constitute the core of a moral consensus. Clinicians should preserve the public and the donors’ trust by ensuring the donors’ competence to make decisions and understand the risks, the voluntariness of their decisions, and their motives [21]. To meet their duty of non-maleficence, physicians and transplant coordinators are required to apply subjective moral standards that refer to the belief that morality is based on individual or cultural perspectives, rather than being universally applicable. This means that what is considered right or wrong can vary depending on personal beliefs, experiences, emotions, or societal norms [22]. Thus, it is expected that all members of multi-professional nephrology teams following these subjective moral standards will evaluate the risks involved in kidney donation, taking into account the donor’s age, the genetic background of their family, underlying diseases of their parents, their current state of health, and socio-demographic factors.

### 1.2. The Associated Risks and Long-Term Consequences of Kidney Donation

Living donors must be provided with clear and comprehensive information as to both the short- and long-term outcomes of their donation. This transparency is essential to facilitate informed decision-making, ensure proper follow-up, and guarantee appropriate care [23]. Despite being regarded as a safe procedure with a low risk, living kidney donation carries a lifelong potential for significant long-term risk of ESRD in later life. After donor nephrectomy, compensatory hyperfiltration occurs in the remaining kidney, such that the net reduction in the glomerular filtration rate (GFR), early after donation, is only ~30% (25–40%; i.e., decrement in GFR of 25–40 mL/min per 1.73 m^2^) [23].

Furthermore, a 7.6-year study in the United States and a 15.2-year study in Norway showed that the incidence of ESRD was eight times higher amongst donors than amongst matching healthy non-donors in the control group [24]. Potential immediate complications following a live kidney donation are a reduction in GFR, i.e., an indicator of risk to the kidney, and an adverse effect of a slight rise in blood pressure occurring in up to 10% of cases [25]. Moreover, at least two American donors were subsequently placed on the waiting list for kidney transplantation [26]. Short-term clinical outcomes have documented overall mortality rates of 0.031% for nephrectomy [27,28]. Since long-term complications are known to occur amongst 18% of kidney donors, with additional adverse effects worldwide, concerns as to the safety of live kidney donors and non-maleficence are rising, calling for a revisiting of EC [29].

### 1.3. Ethical Aspects of Living Donor Kidney Transplantation

Donors must be fully informed as to the intermediate and long-term consequences of living with a single kidney that is usually less strong, before formulating decisions regarding their donation. Importantly, the donor’s perception of risk may diverge from the clinicians’ assessments [30]. The donors’ autonomous choice is to act in a way that is consistent with their life goals, values, and beliefs [31]. Yet, there may be tension between ethical values, their interpretation, and implementation [7]. The ethical principle of respect for autonomy requires that potential donors be competent, informed, and able to understand the risks of the procedure [32]. The respect for autonomy calls for an informed consent process that accords with the paramount value of ‘Primum non Nocera’ (first, do no harm) [19]. A central ethical prerequisite relates to the legal obligation of disclosure for live organ donations [33].

The disclosure process within the informed consent procedure transcends merely signing a form. It adheres to the principles of respect for donor autonomy and self-determination: donors possess the right to make voluntary decisions concerning their bodies and to consent to undergo medical procedures [19].

Disclosure principles stress the need to assure the donors’ comprehension of their specific potential risks [34]. Donors have the right to determine the extent of risk they are willing to accept [35]. The disclosure process ends with the donor’s consent to donate, free of coercion, while simultaneously being aware of the customized risks and potential specific long-term ramifications, not only based on published information but also based on their specific clinical evaluation [36,37]. Regulations also require that clinicians ensure that donors understand the necessity of future continued monitoring and that they will need to undergo long-term annual follow-ups at the transplant center or hospital nephrology unit [38]. Variations in the content and quality of the disclosure raise concerns that donors undertake potential health risks due to inadequate informed consent processes, thereby involving an infringement of their right to autonomy [39]. Discrepancies in the methodology and substance of the disclosure process can undermine public trust in healthcare professionals, potentially deterring live kidney donations [29].

Accordingly, we explored donors’ satisfaction with the ethical competence of multi-professional nephrology teams regarding the disclosure of donation consequences to live kidney donors.

## 2. Materials and Methods

### 2.1. Study Design and Setting

A cross-sectional study was performed among 91 live kidney donors (>18 years of age) who had donated a kidney in two hospitals that belonged to the Ministry of Health’s Transplantation Center one year after the donation, from December 2018 to December 2020. According to Israel’s Ministry of Health, 1412 Israelis were awaiting kidney transplants by late 2023; of those, 970 were on the transplant list [40]. Amongst 21 developed countries, Israel has the highest rate of kidney failure deaths due to diabetes type 2 [41]. Modern surgical techniques enable kidney removal laparoscopically, thus reducing the risks of open surgery and shortening the convalescence period [42]. Live kidney transplantations offer the best treatment for those with ESRD [43].

### 2.2. Data Collection and Participants

We initiated contact with 108 former living kidney donors via email to gauge their interest in participating in the study; for those who provided consent, a questionnaire was distributed via Google Forms in their native language (Hebrew or Arabic) (n = 62). We also established contact via the telephone with the ultra-Orthodox Jewish donors, who typically refrain from using the internet, to obtain their consent for participation and interviewed them face-to-face in their native language (Hebrew or Arabic) using iPads for data collection (n = 21); in addition, live kidney donors from the Palestinian Authority area who had previously donated a kidney at an Israeli hospital were also interviewed face-to-face in their native language (Arabic) (n = 8).

Our study concentrates on the ethical climate among multi-professional nephrology teams, seeking to derive insights into their EC within the realm of live kidney donations [9,11]. Although studies have documented variations in disclosure processes, few studies have examined the content of the disclosure process in this context [44,45,46,47]. We focused on factors that may compromise the respect for the right to autonomy, the validity of informed consent, and their outcomes. We identified gaps between values and their implementation and facilitated a higher ethical awareness by strengthening EC in personnel teams handling live kidney donations. We concentrated on three questions in the interview with the donors: a. Do multi-professional teams demonstrate EC? b. Does the content of the disclosure process with live kidney donors necessitate respect for their right to autonomy and the disclosure of potential risks for the live kidney donor in both the short- and long-term? c. Are there differences in risk awareness amongst donors?

### 2.3. Study Measure

Disclosure content was assessed using Rodrigue et al.’s [44] evaluation questionnaire, comprising 15 items measuring disclosure elements routinely included in the informed consent process for potential living kidney donors. A single solution factor analysis yielded a score of 0.59 and Cronbach’s alpha of 0.76.

Risk awareness was assessed using Matas et al.’s [48] 10 items routinely used in the screening process for living kidney donors. An index of awareness of the ramifications of the donation was created using six items from Rodrigue et al.‘s instrument, with the following questions: (1) Were you aware of the potential effect of the donation on your ability to obtain life insurance? (2) Were you aware of the potential impact of the donation on your lifestyle (i.e., ability to retain or obtain future employment)? (3) Were you aware of clinical uncertainties (i.e., long-term donor complications; did you know which kidney was to be donated and why?) (4) Were you aware of the ramifications of living with one kidney? (5) Were you aware of the risks of developing kidney renal failure, high blood pressure, microalbuminuria, proteinuria, and hyperplasia? (6) Were you aware that your estimated (eGFR) rate and kidney functioning may decline? The index was categorical (yes = 1; no = 0). Responses for the 10 items were on a five-point Likert scale: “not at all”, “slightly”, “moderately”, “highly”, and “extremely high”. A high index score indicates a higher awareness of life-long risks. We presented the findings by the mean and standard deviations (SD) of the participants’ answers.

Socio-demographic control variables were measured and then transformed into category variables: gender (male; female); age at donation (18–29; 30–39; 40–49); schooling years (up to 13 years; academic); marital status (single/divorced; married).

### 2.4. Statistical Analysis

A Kolmogorov–Smirnov test was performed to examine the normal distribution. The study sample was described using means, standard deviations (SD) for continuous variables, and numbers and percentages for categorical variables. Differences in creatinine, BMI, and GFR levels between live kidney donors before and one year after donation were examined using a paired sample *t*-test. A two-sided *p* value < 0.05 was considered statistically significant. Data were analyzed using SPSS version 28 (IBM, Armonk, New York, NY, USA).

### 2.5. Ethical Considerations

The study protocol was approved by the Institutional Review Board (IRB) at the Academic College of Tel Aviv-Yaffo (protocol number 2018-2018015; approval date: 11 December 2018). All procedures were performed following local guidelines and regulations. The participants signed an informed consent form, authorizing their participation in the study, as well as the publication of its findings.

## 3. Results

Overall, 91 live kidney donors aged 18–49 years were enrolled. Of those, 65.9% were males, 54.9% were academic participants, 76.9% had three children or above, 86.8% were employed, and 76.9% were married. Among the live kidney donors, 59.3% reported that the motivation behind the donation was a first-degree family member vs. 35.2% altruistic and 5.5% commercial (Table 1).

Table 2 displays the attitudes of kidney donors toward the ethical aspects of nephrology staff. Only 13.2% reported that the provided disclosure adequately explained the possible consequences of living with a single kidney. Since a right-side kidney donation is linked to a higher occurrence of technical organ rejection (i.e., a short renal vein and a higher risk of narrowing and thrombosis of the renal veins) [49], 72 of the 91 donors had their stronger left kidney removed. They were not told that the left kidney is the bigger and more effective one and that due to the increased workload on the remaining right kidney, it might grow beyond its normal dimensions after the transplant, potentially causing ESRD. Most donors reported that they had no scheduled follow-up examinations as required; however, 77.8% reported that they received an explanation from a nephrologist at the Transplantation Center and were referred to their general practitioner for monitoring. Approximately 20% of the participants reported that the disclosure included information regarding their risk of developing ESRD, hypertension, and proteinuria, whereas 23% of the participants reported that the disclosure covered the potential for reduced kidney function within a year post-donation as a post-donation illness or complication. Disclosure regarding which kidney would be removed was reported by 83.5% of the participants (Table 2).

Table 3 displays donors’ attitudes toward the ethical competence of the nephrology staff in the explanation and disclosure of potential risks before donation and the follow-up after donation. The donors reported a low mean of the index score that indicates a low follow-up by the physician after the donation (mean = 1.16, SD = 0.37), a low extent of explanations about who would monitor their recovery from the surgery (mean = 2.35, SD = 0.90), and a low extent for the follow-up conducted, as explained to the participant before the donation (mean = 1.33, SD = 0.47). However, the donors reported moderate to high concerns about the short- and long-term risks associated with the donation (mean = 3.03, SD = 0.87) or life after organ donation (mean = 3.87, SD = 0.82) (Table 3).

The mean level of creatinine was significantly higher in the post-donation period one year following a kidney donation (1.2 mg/dL) than in the pre-donation period (0.9 mg/dL), *p* < 0.001. However, the mean GFR level was significantly lower in the post-donation period one year following a kidney donation (117.8 mL/min/1.73 m^2^) compared with the pre-donation period (84.0 mL/min/1.73 m^2^), *p* < 0.001 (Table 4). The donor’s BMI level increased one year after donation compared to the pre-donation period, with a significant difference between the pre- and post-donation period (25.9, 26.8, respectively), *p* < 0.001, although it was controlled during the pre-donation period (Table 4).

## 4. Discussion

The practice of living kidney donation is grounded in the ethical principles of beneficence and non-maleficence, which advocates for significant benefits to the recipient while ensuring that the risks to the donor are justifiably minimal. Moreover, it emphasizes the autonomy of the donor, predicated on the provision of informed consent, where all the potential risks and uncertainties are thoroughly disclosed. Both donors and their healthcare providers must have a comprehensive understanding of the risks associated with kidney health, surgical complications, and psychosocial outcomes. This study makes several contributions. It aims to bridge the gap in existing knowledge by examining the ethical conduct of multi-professional nephrology teams in interactions with live kidney donors, specifically assessing the respect for donors’ autonomy and the extent of information disclosure during the informed consent process. Methodologically, unlike previous studies that were limited by ethnic homogeneity and overlooked the impact of socioeconomic factors, access to healthcare, and levels of education, this study, despite its limited sample size, explored differences in risk awareness amongst diverse populations with varying socioeconomic statuses and educational backgrounds. Practically, it offers recommendations on ways to enhance ethical conduct in this context.

### 4.1. Disclosure Content and Upholding the Autonomy Rights of Live Kidney Donors

Despite the imperative to honor the autonomy of live kidney donors, the disclosure process was found to be insufficient and, in many instances, negligent, thereby obscuring the potential medium-term implications of donation. Given the recognized potential adverse effects on young donors in the medium and long term, the lack of comprehensive disclosure not only signifies the nephrology team’s failure to conscientiously prevent harm to donors but also restricts the donors’ freedom. Most of the donors reported that they were not fully informed as to the medium- and long-term consequences and the necessity for future monitoring. Under such circumstances, prioritizing the well-being of kidney recipients does not adhere to the principle of utility for young live kidney donors, as it fails to ensure a balance between risks and benefits for these donors. It is conceivable that in their effort to benefit recipients, nephrology teams may have underestimated the risks of hypertension and hypertensive nephropathy in live kidney donors [50].

Although inadequate disclosure might originate from a place of beneficence and may not be deliberate, it nevertheless violates the live kidney donor’s right to autonomy. True respect for the autonomy of donors encompasses actionable respect, not merely a respectful attitude. It is conceivable that the team exhibited soft paternalism, prioritizing what they perceived as the donor’s best interests, potentially justified by the intent to benefit the kidney recipient. This form of soft paternalism could have inadvertently limited the younger donors’ ability to render autonomous choices, as the incomplete, standardized disclosure provided might have influenced the preferences and decisions they would have otherwise made with full information [51]. Such paternalistic conduct may have led to a wrong infringement of donors’ rights and respect for their autonomy, a prima facie condition that was not fulfilled. Appropriate full disclosure may balance out the wrong prima facie evidence. Without this, the infringement of the donors’ right to autonomy would be a pro tanto wrong and morally indefensible. If the participants had been provided with comprehensive information adhering to the professional standard of disclosure and the reasonable person standard of disclosure, it is possible they would have been less confident about deciding to donate their kidney.

#### 4.1.1. Disclosure of Short- and Long-Term Potential Risks to Live Kidney Donors

Despite the clinicians’ important role in providing clinical guidelines for follow-up and supporting the process of informed consent, a reduction in glomerular filtration rate is a known consequence of kidney donation for the short-term; however, the middle- and long-term health risks remain uncertain [52,53,54]. Living donors undergo risks such as potential longer-term consequences of nephrectomy [55]. A systematic review and meta-analysis found that living kidney donors had a higher diastolic blood pressure, poorer renal function, and a higher risk for ESRD than nondonors. Female donors also had a higher risk for pregnancy-related complications, such as preeclampsia [56].

It is conceivable that in an attempt to reconcile the principle of beneficence towards the recipient of the donor’s autonomy, nephrology teams might have made risk assessments based on their perspectives of sacrifice and risk. This approach potentially overrides the donors’ rights to assess and understand the risks according to their perceptions. Notably, donors’ irrational risk perceptions should also be acknowledged as a facet of their autonomy, underscoring the complex dynamics between informed consent, ethical medical practice, and respect for donor autonomy [57].

Nephrology teams prioritize the welfare of the kidney recipient, disregarding the preferences of a young donor by rationalizing such actions through an appeal to the principle of beneficence towards the recipient, thus undermining the validity of the donor’s consent to the procedure [58]. To safeguard the well-being of a potential kidney recipient, for instance, through harm prevention, harm removal, and the promotion of positive outcomes, it is imperative that nephrology teams deliberately refrain from engaging in practices that could result in future detriment to the donor. This approach is rooted in the principle that the duty to not harm (non-maleficence) holds greater weight and takes precedence over the obligation to do good (beneficence) [59]. In light of the expanding and changing nature of the living donor population, together with advancements in surgical techniques, it is crucial to monitor the short- and long-term risks associated with a living kidney donation. This monitoring is essential for facilitating genuinely informed consent and preserving public confidence in living kidney donations. The transplant community must continue its endeavors to precisely evaluate risks amongst living kidney donors from a wide range of demographic backgrounds.

#### 4.1.2. Variations in Risk Perception Amongst Donors Based on Demographic Characteristics

Although the current estimates do not account for the pertinent risks of ESRD in young donors, whose life expectancy may be >60 years old [23], our findings indicate that within just one year post-donation, young donors exhibited an elevated risk of ESRD later in life. Earlier research highlighted a moderate increase in urine protein levels and elevated blood pressure as consequences of kidney removal [60,61]. Moreover, 24% of the participants encountered complications. It is conceivable that the genetic predispositions of other participants might predispose them to health risks and a diminished quality of life in their middle and later years. Donors were not fully cognizant of these potential consequences. Furthermore, follow-up examinations were often brief and predominantly conducted by general practitioners, who may not specialize in nephrology.

The findings reveal that the vast majority of donors were below the age of 40, a demographic for which kidney donation has been linked to a significant compensatory increase in the estimated GFR in the remaining kidney, reaching ~70% of the pre-nephrectomy levels [62,63]. The level of awareness as to potential consequences differed across demographic groups. Secular donors tended to underestimate the significance of follow-up care, possibly due to a lack of awareness concerning the risks associated with the absence of a follow-up. Awareness levels were notably higher among women, employed individuals, and donors residing in major urban areas. These findings underscore concerns as to EC and emphasize the imperative to nurture such competence within multi-professional teams engaging with donors.

### 4.2. Enhancements to Address Deficiencies in the Disclosure Process Content

The foundation for sustainable care is anchored in the articulation and application of explicit ethical values [64]. A donor, motivated by the profound emotional fulfillment derived from saving another’s life, should not be subjected to physical detriment [61]. Moreover, the increasing instances of living kidney donations may reflect the esteem in which donors and the broader public hold medical professionals, recognizing their dedication and virtuous contributions. To reclaim trust in clinicians, nephrology teams must employ virtue-based EC [20]. The findings call upon multi-professional nephrology teams to apply a subjective moral standard. The use of the online risk tool in kidney donor acceptance protocols is essential to minimize the number of living kidney donors in whom ESRD develops, as well as to support donation in individuals whose long-term risk was previously misunderstood and help validate their informed consent [23].

Our findings advocate for teams to exercise caution and adhere to strict ethical practices when dealing with donors from specific demographics, including younger individuals, males, secular individuals, and the unemployed. Additionally, we emphasize the importance of structuring the disclosure process through the implementation of a comprehensive form that encapsulates all the necessary content. The data indicate inconsistencies in understanding the process and a lack of transparent disclosure of post-donation medical consequences, highlighting the need to improve communication. This underscores the necessity for more standardized and thorough disclosure methods to enhance ethical standards and communication practices, which are the core focus of our research. Previous studies have identified similar and different issues in donors’ experiences and perceptions and have documented the challenges and variations in informed consent processes at different transplant centers [44,45].

Both the clinician and the donor are required to endorse the disclosure form for each element discussed. Nephrology teams must monitor regular short-term and long-term follow-ups for all live kidney donors to identify any post-donation health risks. Furthermore, inadequate ethical competence, particularly as to the principles of beneficence and non-maleficence, demands rigorous supervision to ensure adherence to ethical standards.

#### Enhancing the Ethical Competence of Multi-Professional Teams Working with Kidney Donors

Implementing the EC model for multi-professional teams necessitates an exploration of the reflections of young donors, ensuring the avoidance of soft paternalism and the preservation of their autonomy. The ‘time-to-talk’ aspect highlights the need for ethical discussions among team members, advocating for comprehensive disclosure to donors, whether this communication is facilitated by a nurse, physician, or psychologist involved in the care of kidney donors and recipients [65].

The practice of living donation relies on the ethical principles of beneficence and non-maleficence (where the benefits to the recipient are great, and the risks to the donor are justifiably lower), as well as donor autonomy after informed consent (where the risks and uncertainties are fully disclosed). Living donors must be provided with clear and comprehensive information regarding both the short-term and long-term outcomes of their donation. This transparency is essential to facilitate informed decision-making, ensure a proper follow-up, and guarantee appropriate care [66,67].

Understanding the risks related to kidney health, the surgical complications, and psychosocial outcomes is vital for donors and their care providers [48,68,69,70,71]. Defining knowns and unknowns can assist in future research and policy development priorities. However, the focus here on these key outcomes, which are important to donors, is not intended to be comprehensive, and other outcomes (e.g., pregnancy risks, hypertension, metabolic conditions, and effects on relationships, family dynamics, and lifestyle) are discussed elsewhere [72]. Some adverse outcomes may only be fully appreciated after decades of follow-ups of large cohorts of donors with diverse demographic characteristics. An ongoing commitment to strengthen the evidence base for donor candidate evaluation, selection, and counseling, including a longer follow-up in representative cohorts, the well-designed use of integrated data sources, long-term registries [73], and the advancement of tools for tailored risk estimation [74], should remain a leading priority for researchers, practitioners, and policymakers.

The cornerstone of the ethical framework that guides the practice of medical professionals is encapsulated in the principle “First, do no harm”. This fundamental tenet highlights the paramount duty of clinicians to prevent any form of harm to donors. It is the responsibility of healthcare practitioners to rigorously observe their professional code of ethics, mandating the provision of relevant information to donors. This duty requires healthcare providers to take into account the donor’s preferences and their ability to grasp the explanations given to them. Furthermore, healthcare professionals must supply exhaustive information regarding diagnoses, treatment plans, and potential outcomes, which includes a thorough discussion of the associated risks, expected prognosis, and the availability of alternative treatment options. This includes an explanation of possible psychosocial effects. For example, while Rodrigue et al.’s [75] study evaluating psychosocial variables both before and after donation demonstrated that donors exhibited minimal to negligible dysfunction in various domains of psychological well-being, donors who had pre-existing concerns were at an elevated risk of encountering similar issues after the donation; other studies have shown psychological issues such as regret and depression post-donation [76]. The objective is to empower donors with the knowledge necessary to make well-informed decisions concerning their treatment, enabling them to either consent to or decline the recommended medical interventions, grounded in a comprehensive understanding of their choices.

Multidisciplinary nephrology team management should be encouraged to shape a culture of ethics, with values that guide the entire multi-professional team towards a shared way of thinking as regards the values in practice, both as teams and as individual clinicians, to design a disclosure process that takes into consideration donors and to create an internal value hierarchy to assess what is best for each donor [77,78,79]. Such a multi-professional team could benefit from the contribution of mental health professionals, such as a social worker and a psychologist. Previous studies highlight the need for comprehensive donor education and ethical competence among nephrologists and all members of the transplant teams [80,81]. In an ethical culture, values and their implementation with donors who are treated with dignity and respect are to be routinely monitored, despite the distress of the recipients. Leuter et al. call for training in EC to improve the capacity of multi-professional nephrology teams to manage ethical issues regarding donors [82].

Training should focus on acquiring reflection skills and considering the teams’ values, goals, and accountability toward donors. We believe that to strengthen EC, certain interventions should be performed: (a) practicing the skill of reflection to ascertain one’s reactions, becoming conscious of oneself in the team, gaining insights into one’s EC; (b) reflecting with team members, thus standing together as a team, providing collegial support in difficult ethical situations; (c) holding routine discussions on the full disclosure process with the donor; (d) practicing one’s listening skills to understand each other’s viewpoints; (e) reviewing what has been done well and what has not worked; (f) talking about ethical dilemmas relating to donors to raise awareness and involvement and reduce automatic actions; (g) working towards common goals and creating a shared ethical value base, particularly towards young donors.

We recommend explaining to potential donors the potential long-term health ramifications following a kidney donation, to maintain the ethical principles of beneficence and non-maleficence and to disclose to the donor all the possible risks and complications in the remaining organs, including the residual kidney, as part of the informed consent process. Hence, rigorous and comprehensive post-donation monitoring and follow-up by both primary healthcare providers and nephrology specialists are essential to mitigate and manage potential adverse outcomes effectively, thus ensuring the well-being of the donors after the donation and over the long term.

As transplant coordinators and nephrologists, we bear a responsibility for diligently overseeing the health of individuals who have undertaken considerable risks and made substantial sacrifices by donating to benefit others. Our team is acutely aware of the potential long-term health implications that organ donations can have on a donor’s health and overall quality of life. While we commend donors for their altruistic contributions, it is paramount that we sustain proactive engagement and establish open channels of communication by creating a specialized clinic for kidney donors, ensuring that donors adhere to their post-donation follow-up appointments with their primary healthcare providers. In instances where such follow-ups are not supervised properly, we have to inform and collaborate with the attending physicians, thereby guaranteeing the continuation of essential oversight and medical care. Our dedication to donors extends well beyond the act of donation itself, underscoring our commitment to their long-term health and sustained quality of life.

The medical personnel involved in transplant coordination try to ensure that donors undergo the continuous monitoring of renal function following organ donation. Before organ donation, it is critical to engage in comprehensive discussions with potential donors as to the immediate and long-term repercussions of their decision, including considerations related to familial genetics.

It is very important to explain to young donors—particularly those who anticipate family planning in the future, including marriage and childbirth—that the risk of developing renal complications is relatively low in the early years; however, this risk increases after the age of 40, due to heightened susceptibility to conditions such as diabetes and cardiovascular, vascular, and autoimmune diseases.

Understanding the elevated risks of hypertension, preeclampsia, and gestational diabetes as a consequence of kidney donation is essential for women who have not yet conceived or who are contemplating future pregnancies. However, according to the data collected by the United Network for Organ Sharing (UNOS)/Organ Procurement and Transplantation Network, living donors can expect a permanent reduction in kidney function of 25–35% following donation [75]. Such statistics are instrumental in forecasting the lifetime risk of chronic kidney disease or ESRD in these donors.

### 4.3. Limitations of the Study

Our study had some limitations. It had a small number of participants, and needs to be confirmed with a larger cohort study. The generalizability of the findings drawn in the current study is restricted, due to the process varying significantly among transplant centers, leading to confusion, misconceptions, and a lack of motivation among potential donors. Therefore, the donor evaluation, allocation, and post-donation follow-up processes must be standardized globally. The information was obtained by self-reporting from the participants one year after donation, which might have led to recall and reporting bias but with non-differential misclassification. However, this information is usually not available in administrative databases. Significant events such as medical procedures tend to be well remembered by people. Donating a kidney is a major life event, when donors are expected to clearly remember information given to them during the sensitive processing stage [83].

## 5. Conclusions

Our findings display that donors’ satisfaction with the ethical competence of multi-professional nephrology teams regarding the disclosure of donation consequences to live kidney donors is low. The donors reported a low rate of follow-up by the multi-professional nephrology team after the donation, an insufficient explanation about who would monitor their recovery from the surgery, and a low level of satisfaction with the follow-up, which was not conducted as explained to the participant before the donation. Identifying the associated factors with the ethical competence of multi-professional nephrology teams is essential to prevent non-maleficence toward young kidney donors. This study indicated that donors are at an increased risk of worsening kidney function (creatinine and GFR), and BMI. Our findings underscore the imperative to advise donors that their condition may worsen over time and can result in complications; thus, they should be monitored during short and long-term follow-up periods.

## Figures and Tables

**Table 1:** Socio-demographic characteristics of the study participants.

	*N*	%
Gender		
Male	60	65.9
Female	31	34.1
Age at donation		
18–29	13	14.2
30–39	39	42.9
40–49	39	42.9
Schooling years		
Up to 12 years	41	45.1
Academic	50	54.9
Number of children		
0–2	21	23.1
≥3	70	76.9
Employment		
Employed	79	86.8
Unemployed	12	13.2
Marital status		
Single/Divorced	21	23.1
Married	70	76.9
**The motivation behind the donation**		
First-degree family member	54	59.3
Altruistic	32	35.2
Commercial	5	5.5

**Table 2:** Attitudes of kidney donors toward ethical aspects of nephrology staff.

Statements of Ethical Aspects	*N*	%
**Disclosure regarding life with a single kidney**		
Yes	12	13.2
No	79	86.8
**Disclosure regarding the risk of developing end-stage renal disease, hypertension, and proteinuria after donation**		
Yes	18	19.8
No	73	80.2
**Disclosure regarding the increase in kidney functions after kidney donation**		
Yes	21	23.1
No	70	76.9
**Disclosure regarding which kidney will be removed**		
Yes	76	83.5
No	15	16.5
**An appointment with a mental health professional (psychiatrist, psychologist, or social worker)**		
Yes	21	23.1
No	70	76.9
**I received an explanation from:**		
A nephrologist in the dialysis unit	20	22.2
A nephrologist at the Transplantation Center	70	77.8

**Table 3:** Donors’ attitudes toward the ethical competence of the nephrology staff in the explanation and disclosure of potential risks before donation and the follow-up after donation.

Statements of Ethical Aspects	*Mean*	*SD*
To what extent was the process explained to you in depth?	3.44	0.65
To what extent was the explanation of the process understandable to you?	3.23	0.83
To what extent did you have concerns about the risks associated with the donation?	3.03	0.87
To what extent did you have concerns about the risks in life after the donation?	3.87	0.82
To what extent did you understand the follow-up required after organ donation?	2.73	0.62
To what extent did the physician who convinced you to donate a kidney stay in contact with you and follow your tests?	1.16	0.37
To what extent was it explained to you before the donation who will monitor your recovery from the surgery and how?	2.35	0.90
To what extent was the follow-up conducted as explained to you before the donation?	1.33	0.47
To what extent did you feel that you received emotional support throughout the process?	2.29	0.60
To what extent was it explained to you how long you will be monitored by the attending physician?	2.12	0.79

SD: standard deviation.

**Table 4:** Creatinine, BMI, and GFR levels before and post donation.

Kidney Functions (Creatinine and GFR), and BMI Levels	before Donation	One Year after Donation	*p* Value
Creatinine (mg/dL), mean (SD)	0.9 (0.04)	1.2 (0.1)	<0.001
GFR (mL/min/1.73 m^2^), mean (SD)	117.8 (19.6)	84.0 (13.8)	<0.001
BMI, mean (SD)	25.9 (2.6)	26.8 (3.9)	<0.001

SD: standard deviation; GFR: glomerular filtration rate; BMI: body mass index.

## Data Availability

The data presented in this study are available on request from the corresponding author.

## Public Involvement Statement

There was no public involvement in this research.

## Guidelines and Standards Statement

This manuscript was drafted against Strengthening the Reporting of Observational Studies in Epidemiology (STROBE) for the quantitative study and Consolidated Criteria for Reporting Qualitative Research (COREQ) for the qualitative analysis.
